# Training Characteristics, Personal Factors and Coping Strategies Associated with Burnout in Junior Doctors: A Multi-Center Study

**DOI:** 10.3390/healthcare9091208

**Published:** 2021-09-14

**Authors:** Nurhanis Syazni Roslan, Muhamad Saiful Bahri Yusoff, Asrenee Ab Razak, Karen Morgan, Nor Izzah Ahmad Shauki, Anjanna Kukreja, Norashidah Rahmat, Chin Ri Wei Andrew, Muhammad Fikri Shaharudin Basri, Abdullah Shamshir Abd Mokti, Nur Haziyah Md Yazid, Munirah Ismail, Pangie Bakit

**Affiliations:** 1 Department of Medical Education, School of Medical Sciences, Universiti Sains Malaysia, Kota Bharu 16150, Malaysia; msaiful_bahri@usm.my (M.S.B.Y.); mfikri94@yahoo.com (M.F.S.B.); 2 Department of Psychiatry, School of Medical Sciences, Universiti Sains Malaysia and Hospital USM, Universiti Sains Malaysia, Kota Bharu 16150, Malaysia; asrenee@usm.my; 3 Perdana University-Royal College of Surgeons in Ireland School of Medicine, Perdana University, Kuala Lumpur 50490, Malaysia; karenmorgan@perdanauniversity.edu.my; 4 Department of Health Psychology, Royal College of Surgeons in Ireland, D02 YN77 Dublin, Ireland; 5 Institute for Health Systems Research, National Institutes of Health, Ministry of Health, Shah Alam 40170, Malaysia; drizzah@moh.gov.my; 6 Department of Medicine, University Malaya Medical Centre, Kuala Lumpur 59100, Malaysia; anjanna@ummc.edu.my; 7 Department of Pathology, Hospital Sultanah Aminah Johor Bahru, Ministry of Health, Johor Bahru 80100, Malaysia; rashidah_89@yahoo.com; 8 Department of General Surgery, Hospital Queen Elizabeth, Kota Kinabalu, Ministry of Health, Kota Kinabalu 88200, Malaysia; andrew9329@gmail.com; 9 Department of General Medicine, Hospital Kuala Lumpur, Ministry of Health, Kuala Lumpur 50586, Malaysia; ab.shamshir@gmail.com; 10 Hospital Tunku Azizah, Jalan Raja Muda Abdul Aziz, Kampung Baru, Ministry of Health, Kuala Lumpur 50300, Malaysia; hazichang@gmail.com; 11 Institute for Health Management, National Institutes of Health, Ministry of Health, Shah Alam 40170, Malaysia; munirahismail@moh.gov.my (M.I.); pangiebakit@moh.gov.my (P.B.)

**Keywords:** junior doctor, internship, resident, burnout, well-being, resilience, coping, medical education

## Abstract

Physician burnout has been recognized as a public health crisis. However, there is a paucity of burnout studies in the context of medical internship. We assessed the prevalence and relationship between various training characteristics, personal variables, resilience, and coping with burnout in a cross-sectional study involving 837 interns from ten hospitals across Malaysian healthcare system. The instrument package included demographic questions, the Connor–Davidson Resilience Scale, Brief COPE and the Copenhagen Burnout Inventory. A total of 754 (90.1%) interns completed the inventories. We found a high prevalence of personal-related (73.3%), work-related (69.1%), and patient-related (43.4%) burnout among Malaysian interns. Multivariable analysis showed female gender (odds ratio (OR):1.50; 95% confidence interval (CI): 1.02–2.20), prior work experience (OR: 1.56; 95% CI: 1.05–2.30), and irregular spirituality routines (OR: 1.97; 95% CI: 1.30–2.99) were associated with increased odds of personal-related burnout. Irregular spirituality routines (OR: 2.24; 95% CI: 1.49–3.37) were associated with work-related burnout, while living with other people (OR: 1.77; 95% CI: 1.15–2.73) was associated with patient-related burnout. Lower resilience levels and avoidant copings were associated with personal-, work-, and patient-related burnout. Burnout prevalence among interns is high. The findings support the value of individual-targeted alongside organizational-targeted intervention in burnout reduction. As burnout is prevalent in both years of internship training, ongoing burnout prevention and wellbeing measures are deemed necessary.

## 1. Introduction

Burnout can be defined as work-related, multi-dimensional psychological syndrome that can be characterized by exhaustion, depersonalization and a reduced sense of personal accomplishment [1,2]. Recent systematic review reported an alarming burnout prevalence among physicians (up to 80.5%) [3]. Burnout was shown to be more prevalent in physicians at various stages of medical training than in their peers from the general population [4,5].

Burnout can be driven by various long-term stressors such as heavy workload, long hours, required use of electronic medical records, lack of job autonomy and poor interpersonal relationship [6,7]. In a person who experienced burnout, the enthusiasm turned to exhaustion, the fulfilling involvement became cynicism and the efficiency declined to inefficacy [1]. Although very few studies have examined the long-term impacts of burnout, a growing body of evidence have linked burnout with patient care practices (increased medical errors, longer recovery time, reduced patient satisfaction, and reduced perceived safety), organizational performance indicators (reduced productivity, increased turnover and costs) and physician health (increased substance abuse, depression and accidents) [7,8].

On top of the established factors mentioned above, several studies have explored burnout association with medical specialties and length of training experience, giving mixed results [7,8,9,10,11]. Interestingly, although the work environment has been thought of as the main contributor to burnout, some personal characteristics and ‘less-trait’ aspects, such as education, were also found to influence burnout through workload perception [5,12]. Conversely, resilience and spirituality have been increasingly recognized in mitigating burnout through increased insights, self-care and engagement in positive behaviors [13,14]. Studies also suggested that certain coping mechanisms may have positive or negative impacts on physician burnout [14,15,16].

While many studies of burnout focused on medical students, residents and specialized physicians, there have been few studies on medical interns. Internship is an important step in physician’s career where it serves as the transition phase from the protected environment of student years to patient care responsibilities. The phase often coincides with major life milestones such as first job, relationship, childbirth and was found to have the highest attrition as compared to the other training stages [17,18]. Burnout was initially proposed to be a late-career syndrome, but growing evidence suggested that younger physicians are actually more at risk to develop burnout [19]. 

Under the Medical (Amendment) Act 2012, all medical graduates in Malaysia are required to undergo two years of internship training prior to full registration in designated training hospitals [20,21]. During this mandatory training, medical interns or house officers rotate across five compulsory rotations (medicine, surgery, pediatric, obstetrics and gynecology and orthopedic), and one elective rotation (emergency medicine, anesthesiology, primary care or psychiatry). Each rotation spans for four months. Although internship is ideally a formative time of professional development, interns are expected to take increasing responsibility for patient care and equip themselves with appropriate knowledge, skills and attitudes. This overwhelming responsibility may set the stage for interns to develop mental health problems and burnout [22].

A previous study on Malaysian interns in a single institution university hospital using Maslach Burnout Inventory (MBI) revealed a burnout prevalence of 26.5% [23]. Having said that, internship training has since changed to a contract scheme, capped at 60 h per week with a revised assessment system. Some institutions have introduced intern shadowing program in the final year curriculum where medical students are given some responsibility to patient care to improve the work preparedness of the graduates [24]. A study in the United Kingdom found that intern shadowing is associated with a lower anxiety level among interns [25]. However, less is known whether intern shadowing reduce the occurrence of burnout among interns.

Building on the above conceptual framework and lack of recent data, we set out to investigate the prevalence of burnout and its relationship with various training characteristics, and personal variables among interns in Malaysia. We hypothesized that certain training variables (e.g., rotation and length of experience in internship) and personal factors (e.g., gender, marital status, undergraduate training background, previous extracurricular involvement, work experience, spirituality practices, support at home, coping mechanism, and resilience level) have significant associations with burnout.

## 2. Materials and Methods

### 2.1. Study Design

The study utilized a multi-centre cross-sectional design.

### 2.2. Study Participants

During the study conception, there were 11,706 interns in 46 training hospitals. Using stratified cluster sampling [26], we selected a total of ten training hospitals across all six network zones in the Malaysian healthcare system to represent the geographical distributions (Central, North, South, East, Sabah and Sarawak), variation in patient care needs, and Ministry of Health/Ministry of Education training hospitals.

As there was no prior study in the population that measures on burnout, we adopted the sample size calculation formula for a known population size and unknown population standard deviation [27]. However, as the study utilized stratified cluster sampling, a correction factor (design effect of 1.8) was applied to consider for intra cluster correlation effect [28]. After including a non-response rate of 20%, the final required sample size was 837 interns. In order to better represent the intern population, we determined the sample size for each stratum (network zone) based on its stratum size.

### 2.3. Study Tools

To examine the complex array of variables that could contribute to intern’s burnout, we developed the demographic questions based on the conceptual framework discussed above. The survey questions covered personal demographics (gender, marital status, undergraduate academic result, working experience prior to internship, spirituality routine, and available support at home), undergraduate training background (local versus abroad, intern shadowing, and extracurricular involvement) and training demographics (current rotation and length of experience through internship). As Malaysia is a multi-racial country [29], we gave some descriptors of regular versus irregular spirituality routines based on several major religions professed in the country. 

The survey also included 3 validated instruments: Connor–Davidson Resilience Scale (CD-RISC) [30], Brief COPE [31], and the Copenhagen Burnout Inventory (CBI) [32]. The ten-items CD-RISC is an instrument to measure resilience and has been validated with Cronbach’s alpha coefficient of 0.85 [30]. However, our pilot study of a sample of Malaysian interns failed to verify the ten-items model but provided an acceptable model fit for a nine-items/one-construct model with reliability Cronbach’s alpha coefficient of 0.92. Thus, we performed the analysis using nine-items version with answers scored on a scale of 1 to 5, where a higher score indicates greater resilience.

Brief COPE is a 28-items inventory that measures 14 coping strategies with answers scored on a scale of 1 to 4. It has been validated with the average subscale reliability coefficients of 0.68 [31]. A recent Brief COPE study among caregivers proposed four dimensions of coping: problem-solving, positive thinking, social support, and avoidance [33]. As avoidant coping has been correlated with burnout [14,16], we focused the analysis using ten items of avoidant coping that measure behavioral disengagement, denial, self-blame, self-distraction, and substance abuse. We treated the scores dichotomously where subdomain score of less than 4 were considered as less-utilized coping strategies while score ranging from 5 to 8 were taken as commonly used coping strategies.

The CBI is a freely accessible tool in the public domain that assesses burnout using strong psychometrics. In contrast to MBI [1,7], CBI regards fatigue and exhaustion as the core components of burnout [32]. The CBI specifies its measurement to three spheres of participants’ life that are attributed to exhaustion: personal-related (six items), work-related (seven items) and patient-related (six items) with reliability coefficients of 0.85 to 0.87 for each subscale. Each item is rated on a scale of 0 (never/to a very low degree) to 100 (always/to a very high degree) [7,32]. We treated the continuous scores dichotomously, where an established average score of 50 or above for each subscale was treated as burnout [34].

### 2.4. Data Collection and Ethical Issues

The data collection took place between 20 July 2018 to 19 January 2019. We performed paper-based data collection as the survey took an average of 20 min to fill in and an online survey of similar length has been shown to have a 20–40% additional rate of dropout [35]. We obtained the names of interns working in the participating hospitals via clinical unit or through each department that conducts internship training. We approached the head of interns from each department and scheduled several sessions to suit both day and night shifts. Several data collection sessions were conducted outside interns’ working hours to accommodate the intern’s schedule. The data collection session was conducted by the primary researcher or a research assistant neither whom had any working authority or supervisory role on the interns. Participation was completely voluntary, anonymous and written informed consent were obtained from all participants. There were no supervisor or hospital authorities that were present during the session and no identification were made for any interns who refuse participation. All participants received an honorarium of 10 dollars as a token of appreciation for their participation. 

### 2.5. Statistical Analysis

For primary analysis, we used descriptive statistics to determine overall burnout prevalence for each subscale and to evaluate prevalence based on each demographic characteristic and on avoidant coping strategies. We used simple logistic regression to examine the relationship of burnout with each variable. For multivariable analysis, we performed binary logistic regression to predict the effect of independent variables on each subscale of burnout. We detected no collinearity for any independent variables, and no variables were removed from the multivariable analysis except for rotation characteristics. All statistical analyses were performed using SPSS version 27 (IBM Corp., Armonk, NY, USA).

## 3. Results

Out of 837 invited participants, 754 (90.1%) completed the inventories. The non-response rate can mainly be attributed to scheduling issues. The mean age of the participants was 26.6 ± 1.4 years. Characteristics of the participants corresponds to the studied population in terms of zone distribution. Participants were less likely to be male (36.3%) and this followed the national profile that reported 60% of Malaysian physicians are female [36]. A total of 53.7% participants were in the first year of training, while 46.3% were in the second year. Fewer participants (4.2%) came from anesthesiology rotation, as it was an alternative rotation to emergency medicine. Other demographics distribution can be found in Table 1.

### 3.1. Prevalence of Burnout among Interns

As illustrated in Table 2, the overall prevalence of burnout for Malaysian interns were 73.3% (personal-related), 69.1% (work-related) and 43.4% (patient-related). Personal- and patient-related burnout were highest for interns in emergency medicine rotation (79.0% and 54.3%), while work-related burnout was highest for interns in Medicine rotation (76.2%). Anaesthesiology interns had the lowest prevalence of burnout across all three domains. Personal- and work-related burnout were higher in first-year interns while patient-related burnout was higher in second year interns. Personal-, work-, and patient-related burnout were found to be more prevalent in certain characteristics (female, married, “pass” results in undergraduate exam in contrast to honours, no extracurricular involvement in undergraduate years, working experience prior to internship, irregular spirituality routines and living with someone at home). Participants who had their undergraduate training at local institutions or who had completed no intern shadowing were found to have a higher prevalence of burnout across the three domains. Burnout was also found to be more prevalent among participants who used any kind of avoidant coping.

### 3.2. Relationship between Training Characteristics and Burnout

As illustrated in Table 3, we found no significant relationship between length of experience in internship and burnout. However, emergency medicine interns were more likely than others to have patient-related burnout (54.3% versus 42.1%; OR: 1.64; 95% CI: 1.03–2.60), while anesthesiology interns were less likely to experience patient-related burnout (21.9% vs. 44.3%; OR: 0.35; 95% CI: 0.15–0.82).

### 3.3. Relationship between Personal Demographics and Burnout

Interns who had irregular spirituality routines were more likely than others to experience personal-related burnout (79.8% versus 70.1%; OR: 1.69; 95% CI: 1.18–2.43) and work-related burnout (77.5% vs. 64.9%; OR, 1.86; 95% CI, 1.32–2.64). No other significant relationship was found for personal- and work-related burnout. Interns who lived with someone at home and interns who had no extracurricular involvements in their undergraduate years were more likely than others to experience patient-related burnout (45.4% versus 34.8%; OR: 1.56; 95% CI: 1.06–2.28 and 47.8% versus 39.8%; OR: 1.38; 95% CI: 1.04–1.85, respectively). We found no significant relationship between gender, marital status, undergraduate results, or prior work experience with any domain of burnout.

### 3.4. Relationship between Undergraduate Training Background and Burnout

We found no significant relationship between undergraduate training background and intern shadowing with burnout.

### 3.5. Relationship between Resilience and Burnout

The mean CD-RISC score in this sample was 31.75 with a standard deviation of 6.39. Consistent with the conceptual framework, we found that with one-point increase of CD-RISC score the odds ratio of burnout decreased for personal-related burnout (OR, 0.89; 95% CI, 0.86–0.91), work-related burnout (OR, 0.88; 95% CI, 0.85–0.90), and patient-related burnout (OR, 0.90; 95% CI, 0.88–0.93).

### 3.6. Relationship between Avoidant Coping and Burnout

Interns who utilized avoidant coping, such as behavioral disengagement, denial, self-blame, and self-distraction, were 1.62 to 7.73 times more likely than others to experience all three domains of burnout (Table 3). Interns who utilized substance abuse coping were 7.27 times more likely than others to experience patient-related burnout (84.2% versus 42.3%; OR: 7.27; 95% CI: 2.10–25.17).

### 3.7. Multivariable Analysis of Burnout

Significant relationships are shown in Table 4. Through multivariable analysis, we found that self-blame (OR, 2.63; 95% CI: 1.66–4.19), self-distraction (OR, 1.99; 95% CI: 1.37–2.89), irregular spirituality routines (OR, 1.97; 95% CI: 1.30–2.99), prior work experience (OR, 1.56; 95% CI: 1.05–2.30), being female (OR, 1.50; 95% CI: 1.02–2.20), or having a lower resilience score (OR, 0.89; 95% CI: 0.86–0.92) were independently associated with personal-related burnout. Behavioral disengagement (OR, 3.24; 95% CI: 1.06–9.88), self-distraction (OR, 2.04; 95% CI: 1.42–2.95), self-blame (OR, 1.99; 95% CI: 1.30–3.06), irregular spirituality routines (OR, 2.24; 95% CI: 1.493.37), or a lower resilience score (OR, 0.88; 95% CI, 0.85–0.90) remained independently associated with work-related burnout. Substance abuse (OR, 4.09; 95% CI: 1.06–15.78), denial (OR, 3.01; 95% CI: 1.55–5.83), self-distraction (OR, 1.71; 95% CI: 1.21–2.40), living with someone at home (OR, 1.77; 95% CI: 1.15–2.73), or having a lower resilience score (OR, 0.91; 95% CI: 0.88–0.93) were independently associated with patient-related burnout.

Table 4 also reports the Nagelkerke R^2^ value that measured the proportion of variance in the proposed models associated with the independent variables. For personal-related burnout, the model explained 23.8% of the variance and correctly identified 75.9% of burnout cases. As for work-related burnout, the model explained 28.4% of the variance and correctly identified 74.1% of burnout cases. While for patient-related burnout, 20.1% of the variance and correctly identified 67.0% of burnout cases.

## 4. Discussion

In this multi-center study, we made several important findings. First, the prevalence of intern burnout was high in all domains. This can be explained by Karasek Job Strain model, which posits that interns are vulnerable to develop burnout due to heavy workload and having little control over their jobs [37]. We found that personal- and work-related burnout prevalence (73.3% and 69.1% respectively) were higher than those in other studies conducted with interns using similar instruments in Australia (36.0% and 31.0% respectively) and India (64.1% and 40.0% respectively) [38,39]. Our patient-related burnout (43.4%) was lower than that in the study from India (68.6%) [38] but higher than that found in a single-institution study involving Malaysian medical residents (25.2%) [40]. Despite stricter implementation of work hour restrictions in the training, we found that the prevalence of burnout in the current study was notably higher than in the previous study [23]. This further supports the concept that long hours are not the sole factor contributing to burnout, and future intervention should address more areas on top of work hours restrictions [10]. 

In terms of training characteristics, our findings were consistent with recent studies that showed Emergency Medicine physicians were at higher risk to develop burnout [41,42]. Some studies reported a significantly higher burnout prevalence among junior physicians [5,11,34,43,44,45] while other studies found no difference [10,46]. Previous work in Malaysia proposed that burnout was highest in the first six months of internship [10], however our findings suggested that burnout was high in both years of training. In Malaysia, interns move to a new rotation every four months, and these lateral moves require them to learn different sets of competencies and adapt to new environment, perpetuating them to cycles of burnout [19]. 

Some studies have suggested that gender is a weak predictor of burnout, while other studies reported no difference in burnout due to gender [1,7]. Through multivariable analysis, we found that female interns were more likely than males to develop personal-related burnout, and this seemed to be consistent with two recent studies of interns in the United Kingdom and Australia [34,47]. We did not find any association between marital status and undergraduate academic results with burnout by either univariate or multivariable analysis. The findings on marital status indifferences corroborate with a study from Iran [48]. However, there was scarce literature that discussed academic performance and physician burnout. 

In Malaysia, about half of the interns had their undergraduate training abroad and faced acculturation to the local healthcare system during their internship. Despite working international medical graduates working in an unfamiliar environment, previous studies from the United States proposed that they suffered less fatigue and burnout [44,49]. Interns who graduated abroad persevered challenges in different healthcare environment as students. Additionally, more medical schools now incorporate intern shadowing programs to address burnout by facilitating the transition to the work environment [50]. We assessed the relationships of undergraduate training background and intern shadowing with burnout, and, contrary to our hypothesis, we found no significant correlations.

We hypothesized that engagement in activities that enhance interpersonal skills would have a protective effect against burnout. Consistent with another study among undergraduate students [51], we found that involvement in extracurricular activities reduced the risk of developing patient-related burnout. In contrast, through multivariable analysis, we found that interns who had working experience prior to internship were more likely to develop personal-related burnout. While we found no literature explaining this, a study on residents found no association between profession building activities with burnout development in residents [14].

Similar to previous studies in residents [14,52], we found that regular spirituality routines lowered the risk of developing personal- and work-related burnout among interns. Spirituality routines have been increasingly recognized as a source of comfort and hope during adversities and have been positively correlated with job commitment [53]. Social support at work has been linked with burnout reduction [1,44]. While several studies found no link between social support at home with burnout [10,14,48], our findings revealed that interns who lived with someone at home were at higher risk to develop patient-related burnout. Work-life balance is recognized as one of the major stressors among physicians [7,18]. Although a family, spouse or housemate could function as a resource of support, they could also be a source of emotional strain for interns [1]. Another possible explanation is that interns spend more time trying to improve their competency, studying for assessment or recuperating from their work, thus struggling to spare some time with family members, spouse or housemates. This could contribute to exhaustion and detachment from patients and should be part of the assessment in evaluating intern burnout. 

Our data also showed that interns who had higher resilience level experienced less burnout. This further supports previous studies that highlighted the negative correlation of resilience with burnout [54,55]. Resilient physicians were shown to generate positive resource spirals through self-care, reflection, and active engagement with their own limitation [55].

The most striking findings in this study was the significant relationship between avoidant coping strategies with all domains of burnout. We found that 63.8% interns utilized self-distraction as one of their coping mechanisms and they were twice as likely to develop burnout. The findings were also in agreement with similar studies conducted with other regions [14,56]. As coping strategies explained 5.4% of the variance in burnout development [56], our data further supports the value of individuals-targeted intervention alongside organization-directed intervention [7,57]. Described individuals-targeted intervention includes stress management, self-care efforts, coping, and mindfulness training [7,58]. Educational intervention such as coping skills training have shown some effectiveness in enhancing resilience [59] and should be integrated in the personal development programs. 

The study provides some important findings that may guide curriculum developers in rethinking the focus of medical training in enhancing the work preparedness of graduates. We echo the call of the Accreditation Council for Graduate Medical Education for internship training programs to focus on well-being [60]. Support at home may not be available or as effective for some interns. Hence, a formal support system through mentoring or support groups should be made available at the workplace. A useful framework to reduce burnout include the Stanford professional fulfilment model that promotes a culture of resilience and well-being in the workplace through reflective groups and healthy work habits, and enhancement of workplace efficiency by improving teamwork, simplifying paper works and reducing bureaucracy [61]. 

### Limitations of Study

Strengths of this study include the multi-center design that represented the Malaysian intern training landscape, a large sample size and the use of validated instruments. As with other cross-sectional surveys, this study has several limitations. First, we were able to assess only limited personal constructs pertaining to burnout and did not assess for organizational constructs. Second, due to logistic issues, the study utilized convenient sampling, and this might limit its generalizability to a wider context. Third, our study relied on self-reported instruments, and there was potential of reporting bias. Fourth, as the data on coping were treated as dichotomous, certain categories had small numbers of participants resulting in unstable OR with wide CI [62]. Fifth, despite suggesting several significant associations, these relationships do not constitute causal effect. Low resilience levels and avoidant coping strategies could have been the effects of burnout rather than the antecedents. Future studies should include organizational variables (such as work flexibility, support in the workplace and the quality of supervision) or longitudinal studies on individual- and organizational-directed interventions that suit the nature of the interns’ work.

## 5. Conclusions

Our findings indicate a high burnout prevalence among Malaysian interns in both years of the training. Certain rotation characteristics, personal variables, low resilience level and avoidant coping were significantly associated with burnout. Burnout is an occupational hazard and active interventions is necessary to prevent adverse impact to the junior doctors, patient care and healthcare system. 

## Figures and Tables

**Table 1:** Training characteristic, personal demographics and undergraduate training background for the participants enrolled in the study (*n* = 754).

Demographic Characteristics	*n* (%)	Actual Distribution in June 2018 (%)
Zone distribution		
Central	306 (40.6)	4364 (37.3)
North	142 (18.8)	1925 (16.4)
East	134 (17.8)	1873 (16.0)
South	63 (8.4)	1768 (15.1)
Sabah	52 (6.9)	896 (7.7)
Sarawak	57 (7.6)	880 (7.5)
Training characteristics: Rotation		
Medicine	126 (16.7)	
Surgery	136 (18.0)	
Pediatric	85 (11.3)	
Obstetrics and Gynecology	101 (13.4)	
Orthopedic	193 (25.6)	
Emergency Medicine	81 (10.8)	
Anesthesiology	32 (4.2)	
Experience in internship		
Less than 1 year	405 (53.7)	
1 to 2 years	349 (46.3)	
Personal demographics: Gender		
Male	274 (36.3)	
Female	480 (63.7)	
Marital status		
Single	628 (83.3)	
Married	126 (16.7)	
Final undergraduate academic results		
Honors	421 (55.8)	
Pass	333 (44.2)	
Extracurricular involvements in undergraduate years		
Yes	417 (56.3)	
No	337 (44.7)	
Work experience prior to internship		
Yes	510 (67.6)	
No	244 (32.4)	
Spirituality routine		
Regular	501 (66.4)	
Irregular	253 (33.6)	
Living with someone at home		
Yes	613 (81.3)	
No	141 (18.7)	
Undergraduate training background		
Local universities	441 (58.5)	
Abroad universities	313 (41.5)	
Intern shadowing in undergraduate years		
Yes	170 (22.5)	
No	584 (77.5)	

**Table 2:** Burnout prevalence for overall, training characteristics, personal demographics, undergraduate training background and avoidant coping (*n* = 754).

Demographic Characteristics	Personal Related Burnout *n* (%)	Work Related Burnout *n* (%)	Patient Related Burnout *n* (%)
Overall	553 (73.3)	521 (69.1)	327 (43.4)
**Training characteristics**			
Rotation			
Medicine	98 (77.8)	96 (76.2)	57 (45.2)
Surgery	99 (72.8)	92 (67.6)	59 (43.4)
Paediatric	60 (70.6)	56 (65.9)	32 (37.6)
Obstetrics and Gynaecology	69 (68.3)	66 (65.3)	36 (35.6)
Orthopaedic	144 (74.6)	134 (69.4)	92 (47.7)
Emergency Medicine	64 (79.0)	58 (71.6)	44 (54.3)
Anaesthesiology	19 (59.4)	19 (59.4)	7 (21.9)
Experience in internship			
Less than 1 year	298 (73.6)	290 (71.6)	166 (41.0)
1 to 2 years	255 (73.1)	231 (66.2)	161 (46.1)
**Personal demographics**			
Gender			
Male	194 (70.8)	188 (68.6)	115 (42.0)
Female	359 (74.8)	333 (69.4)	212 (44.2)
Marital status			
Single	457 (72.8)	428 (68.2)	264 (42.0)
Married	96 (76.2)	93 (73.8)	63 (50.0)
Final undergraduate academic results			
Honours	307 (72.9)	290 (68.9)	181 (43.0)
Pass	246 (73.9)	231 (69.4)	146 (43.8)
Extracurricular involvements in undergraduate years			
Yes	298 (71.5)	280 (67.1)	166 (39.8)
No	255 (75.7)	241 (71.5)	161 (47.8)
Work experience prior to internship			
Yes	385 (75.5)	361 (70.8)	221 (43.3)
No	168 (68.9)	160 (65.6)	106 (43.4)
Spirituality routine			
Regular	351 (70.1)	325 (64.9)	217 (43.3)
Irregular	202 (79.8)	196 (77.5)	110 (43.5)
Living with someone at home			
Yes	452 (73.7)	426 (69.5)	278 (45.4)
No	101 (71.6)	95 (67.4)	49 (34.8)
**Undergraduate training background**			
Local universities	325 (73.7)	305 (69.2)	196 (44.4)
Abroad universities	228 (72.8)	216 (69.0)	131 (40.1)
Intern shadowing in undergraduate years			
Yes	118 (69.4)	116 (68.2)	66 (38.8)
No	435 (74.5)	405 (69.3)	261 (44.7)
**Avoidant coping**			
Behavioural disengagement			
Yes	60 (90.9)	62 (93.9)	46 (69.7)
No	493 (71.7)	459 (66.7)	281 (40.8)
Denial			
Yes	52 (86.7)	52 (86.7)	43 (71.7)
No	501 (72.2)	469 (67.6)	284 (40.9)
Self-blame			
Yes	210 (86.8)	200 (82.6)	126 (52.1)
No	343 (67.0)	321 (62.7)	201 (39.3)
Self-distraction			
Yes	375 (78.0)	355 (73.8)	229 (47.6)
No	178 (65.2)	166 (60.8)	98 (35.9)
Substance abuse			
Yes	17 (89.5)	19 (100)	16 (84.2)
No	536 (72.9)	502 (68.3)	311 (42.3)

**Table 3:** Relationship between training characteristics, personal demographics, undergraduate training background and avoidant coping with burnout (*n* = 754).

	Crude Odds Ratio (95% CI) *	*p*-Values **
**Personal related burnout**		
Personal demographics		
Irregular spirituality routines	1.69 (1.18–2.43)	0.004
Higher resilience score	0.89 (0.86–0.91)	<0.001
Avoidant coping		
Behavioural disengagement	3.96 (1.68–9.31)	0.002
Self-blame	3.23 (2.14–4.90)	<0.001
Denial	2.50 (1.17–5.37)	0.018
Self-distraction	1.89 (1.36–2.63)	<0.001
**Work related burnout**		
Personal demographics		
Irregular spirituality routines	1.86 (1.32–2.64)	<0.001
Higher resilience score	0.88 (0.85–0.90)	<0.001
Avoidant coping		
Behavioural disengagement	7.73 (2.78–21.52)	<0.001
Denial	3.12 (1.46–6.68)	0.003
Self-blame	2.83 (1.94–4.13)	<0.001
Self-distraction	1.82 (1.32–2.49)	<0.001
**Patient related burnout**		
Training characteristics		
Emergency Medicine	1.64 (1.03–2.60)	0.037
Anaesthesiology	0.35 (0.15–0.82)	0.016
Personal demographics		
No extracurricular involvements in undergraduate years	1.38 (1.04–1.85)	0.028
Lived with someone at home	1.56 (1.06–2.28)	0.023
Higher resilience score	0.90 (0.88–0.93)	<0.001
Avoidant coping		
Substance abuse	7.27 (2.10–25.17)	0.002
Denial	3.65 (2.04–6.53)	<0.001
Behavioural disengagement	3.33 (1.93–5.75)	<0.001
Self-blame	1.68 (1.24–2.29)	0.001
Self-distraction	1.62 (1.20–2.20)	0.002

Abbreviations: CI, confidence interval; OR: odds ratio; 95% CI: 95% confidence interval. * With the exception of resilience score, the odds ratio reflects the ratio of burnout occurring in the described cohort to the ratio of burnout on the other cohort. For resilience score, the odds ratio reflects the ratio of burnout associated with one score increased in CD-RISC instrument. ** Only variables that are statistically significant (*p*-value < 0.05) shown in the table.

**Table 4:** Significant relationship between studied variables and burnout in a descending odds ratio as analyzed from binary logistic regression (*n* = 754).

	Nagelkerke R^2^	Adjusted Odds Ratio (95% CI) *	*p*-Values **
**Personal-related burnout**	0.238		
Avoidant coping: Self blame		2.63 (1.66–4.19)	<0.001
Avoidant coping: Self distraction		1.99 (1.37–2.89)	<0.001
Irregular spirituality routines		1.97 (1.30–2.99)	0.002
Prior working experience		1.56 (1.05–2.30)	0.027
Gender: Female		1.50 (1.02–2.20)	0.041
Higher resilience score		0.89 (0.86–0.92)	<0.001
**Work-related burnout**	0.284		
Avoidant coping: Behavioural disengagement		3.24 (1.06–9.88)	0.039
Irregular spirituality routines		2.24 (1.49–3.37)	<0.001
Avoidant coping: Self distraction		2.04 (1.42–2.95)	<0.001
Avoidant coping: Self blame		1.99 (1.30–3.06)	0.002
Higher resilience score		0.88 (0.85–0.90)	<0.001
**Patient-related burnout**	0.201		
Avoidant coping: Substance abuse		4.09 (1.06–15.78)	0.041
Avoidant coping: Denial		3.01 (1.55–5.83)	0.001
Staying with someone at home		1.77 (1.15–2.73)	0.010
Avoidant coping: Self distraction		1.71 (1.21–2.40)	0.002
Higher resilience score		0.91 (0.88–0.93)	<0.001

Abbreviations: CI, confidence interval; OR, odds ratio; 95% CI: 95% confidence interval. * With the exception of resilience score, the odds ratio reflects the ratio of burnout occurring in the described cohort to the ratio of burnout on the other cohort. For resilience score, the odds ratio reflects the ratio of burnout associated with 1 score increased in CD-RISC instrument. ** Only variables that are statistically significant (*p*-value < 0.05) shown in the table.

## Data Availability

The data presented in this study are available on request from the corresponding author.
